# Macromolecular crowding potently stimulates DNA supercoiling activity of *Mycobacterium tuberculosis* DNA gyrase

**DOI:** 10.1016/j.jbc.2023.105439

**Published:** 2023-11-07

**Authors:** Zifang Deng, Prem Chapagain, Fenfei Leng

**Affiliations:** 1 Biomolecular Science Institute, Florida International University, Miami, Florida, USA; 2 Department of Chemistry & Biochemistry, Florida International University, Miami, Florida, USA; 3 Department of Physics, Florida International University, Miami, Florida, USA

**Keywords:** *Mtb* DNA gyrase, macromolecular crowding, PEG, PVA, molecular dynamics simulation, water activity, excluded volume

## Abstract

Macromolecular crowding, manifested by high concentrations of proteins and nucleic acids in living cells, significantly influences biological processes such as enzymatic reactions. Studying these reactions *in vitro*, using agents such as polyetthylene glycols (PEGs) and polyvinyl alcohols (PVAs) to mimic intracellular crowding conditions, is essential due to the notable differences from enzyme behaviors observed in diluted aqueous solutions. In this article, we studied *Mycobacterium tuberculosis* (*Mtb*) DNA gyrase under macromolecular crowding conditions by incorporating PEGs and PVAs into the DNA supercoiling reactions. We discovered that high concentrations of potassium glutamate, glycine betaine, PEGs, and PVA substantially stimulated the DNA supercoiling activity of *Mtb* DNA gyrase. Steady-state kinetic studies showed that glycine betaine and PEG400 significantly reduced the K_M_ of *Mtb* DNA gyrase and simultaneously increased the V_max_ or k_cat_ of *Mtb* DNA gyrase for ATP and the plasmid DNA molecule. Molecular dynamics simulation studies demonstrated that PEG molecules kept the ATP lid of DNA gyrase subunit B in a closed or semiclosed conformation, which prevented ATP molecules from leaving the ATP-binding pocket of DNA gyrase subunit B. The stimulation of the DNA supercoiling activity of *Mtb* DNA gyrase by these molecular crowding agents likely results from a decrease in water activity and an increase in excluded volume.

*Mycobacterium tuberculosis (Mtb)*, a species within the *Mycobacteriaceae* family, is the causative agent of human tuberculosis (TB) (1, 2, 3). It contains two DNA topoisomerases, topoisomerase I and gyrase (4, 5), which are essential to *Mtb* survival (6, 7, 8). *Mtb* DNA gyrase is a type IIA topoisomerase that transiently cleaves and religates the double-stranded DNA, and introduces (−) supercoils to DNA substrates with the hydrolysis of ATP (9, 10, 11). This enzyme is composed of two different subunits, DNA gyrase subunit A (GyrA) and DNA gyrase subunit B (GyrB), which form an active A_2_B_2_ complex (11, 12). The biochemical properties of *Mtb* DNA gyrase have been extensively studied since it was first purified (9, 13). It has the ability to negatively supercoil relaxed and positively supercoiled (Sc) plasmid DNA, as well as decatenate kinetoplast DNA in the presence of ATP and Mg^2+^ (9, 13, 14). Moreover, *Mtb* DNA gyrase can also relax (−) Sc plasmid DNA at higher concentrations in the absence of ATP (9). *Mtb* DNA gyrase can also form *Mtb* gyrase–DNA cleavage complexes greatly stabilized by fluoroquinolones (FQs). This so-called gyrase poisoning mechanism makes FQs among the most effective and prescribed antibiotics (15, 16). Two FQs, levofloxacin and moxifloxacin, are widely used as anti-TB drugs, usually as the second-line antibiotics for multidrug-resistant TB patients (17, 18). Given its essential role, *Mtb* DNA gyrase is a validated and highly promising target for the development of new antibiotics to treat multidrug-resistant TB, particularly in light of the emerging resistance to FQs in *Mtb* (19, 20).

Previous studies have demonstrated that *Mtb* DNA gyrase exhibits significantly slower enzymatic activity in supercoiling plasmid DNA templates and ATP hydrolysis than *Escherichia coli* DNA gyrase (21, 22, 23). For example, a recent single-molecule study reported that *Mtb* DNA gyrase displayed velocities approximately 5.5 times slower than those of *E. coli* DNA gyrase (23). Moreover, the K_M_ value of *Mtb* DNA gyrase is also higher than that of *E. coli* DNA gyrase (22). Additionally, the supercoiling density of the final Sc DNA products by *Mtb* DNA gyrase is lower than that produced by *E. coli* DNA gyrase (22). The mechanisms behind these differences, however, are not fully understood. Previous studies have also demonstrated that potassium glutamate, spermidine, and bovine serum albumin, often used in DNA supercoiling assays, could stimulate the DNA supercoiling activity of various DNA gyrases, including *Mtb* DNA gyrase (9, 24, 25, 26, 27, 28, 29, 30). The mechanism underlying this stimulation remains elusive. One hypothesis is that this stimulation may arise from the crowding effects caused by these agents. Bacterial cells are extremely crowded, packed with biological macromolecules, such as proteins, RNA, DNA, and polysaccharides (31, 32, 33). For example, the concentration of macromolecules inside *E. coli* cells has been estimated at 300 to 400 mg/ml, occupying 20 to 30% of the total volume (34). Therefore, the kinetics and thermodynamics of biochemical reactions under macromolecular crowding conditions may significantly differ from those in *in vitro* diluted buffer solutions (31, 33). Indeed, *in vitro* studies utilizing buffer solutions that mimic *in vivo* crowding environments, using synthetic polymers such as polyetthylene glycols (PEGs), have shown that macromolecular crowding greatly affects protein stability (35, 36), protein–protein interactions (37, 38), protein–DNA interactions (39, 40), and rates of biochemical reactions (38, 41, 42). However, a systematic and quantitative analysis of the macromolecular crowding influence on DNA topoisomerases, including *Mtb* DNA gyrase, has not been carried out. Understanding these effects is crucial for unraveling the functions of DNA topoisomerases within cells.

In this paper, we conducted a systematic study of *Mtb* DNA gyrase under macromolecular crowding conditions. We discovered that high concentrations of potassium glutamate, glycine betaine, PEGs, and polyvinyl alcohol (PVA) significantly enhanced the DNA supercoiling activity of *Mtb* DNA gyrase. Steady-state kinetic studies demonstrated that glycine betaine and PEG400 decreased the K_M_ of *Mtb* DNA gyrase while simultaneously increasing its V_max_ or k_cat_. This enhancement in *Mtb* DNA gyrase's DNA supercoiling activity can be attributed to the reduction in water activity and the increase in excluded volume under macromolecular crowding conditions. The stimulation of *Mtb* DNA gyrase's DNA supercoiling activity under macromolecular crowding conditions, as unveiled in this study, highlights the crucial role of molecular crowding in modulating the functionality of essential enzymes, offering valuable insights into the complex interplay between biopolymers and enzymes within crowded cellular environments.

## Results

### Stimulating DNA supercoiling activity of *Mtb* and *E. coli* DNA gyrase by potassium glutamate, glycine betaine, PEGs, and PVA

**Figure 1:**
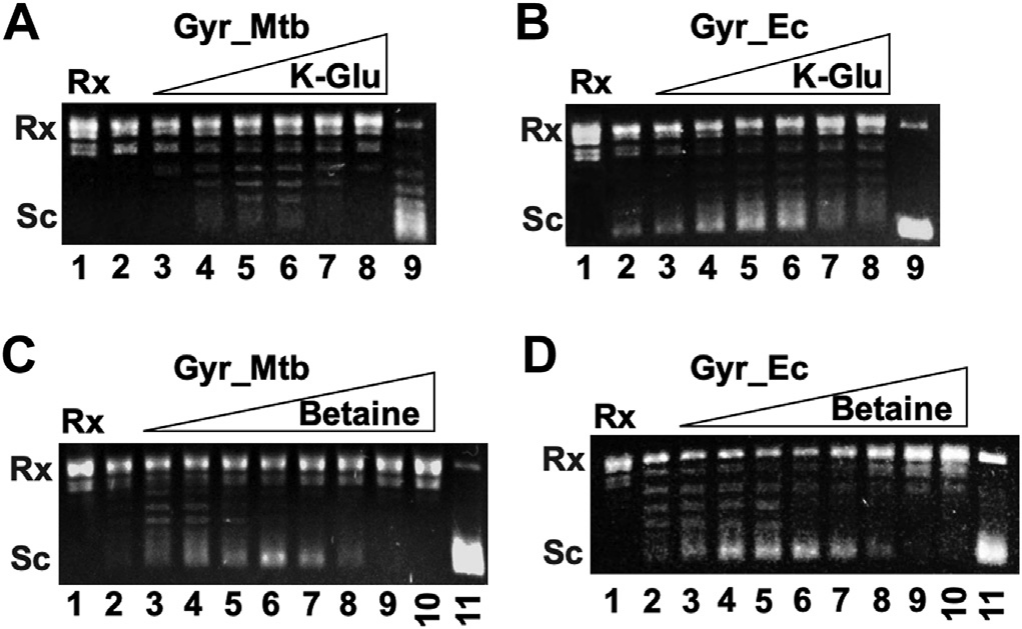
**Stimulation of DNA supercoiling activity of *Mtb* and *Escherichia coli* DNA gyrase by potassium glutamate and glycine betaine.** DNA gyrase supercoiling assays were performed as described under Experimental procedures. Five nanomolars of *Mtb* DNA gyrase or 2 nM of *E. coli* DNA gyrase was used in the assays. The 1× gyrase buffer contains 40 mM Tris–HCl pH 7.5, 10 mM MgCl₂, 100 mM KAc, 4 mM DTT, and 2 mM ATP. *A*, stimulation of the *Mtb* DNA gyrase supercoiling activity by potassium glutamate. Lanes 2 to 8 contain 0, 10, 30, 70, 100, and 200 mM of potassium glutamate, respectively. Lane 1 is the relaxed pAB1. Lane 9 is the DNA sample after the DNA supercoiling assay using 20 nM of *Mtb* DNA gyrase. *B*, stimulation of the *E. coli* DNA gyrase supercoiling activity by potassium glutamate. Lanes 2 to 8 contain 0, 10, 30, 70, 100, and 200 mM of potassium glutamate, respectively. Lane 1 is the relaxed pAB1. Lane 9 is the DNA sample after the DNA supercoiling assay using 20 nM of *E. coli* DNA gyrase. *C*, stimulation of the *Mtb* DNA gyrase supercoiling activity by glycine betaine. Lanes 2 to 10 contain 0, 0.5, 1, 1.5, 2, 2.5, 3, 3.5, 4 M of glycine betaine, respectively. Lane 1 is the relaxed pAB1. Lane 9 is the DNA sample after the DNA supercoiling assay using 20 nM of *Mtb* DNA gyrase. *D*, stimulation of the *E. coli* DNA gyrase supercoiling activity by glycine betaine. Lanes 2 to 10 contain 0, 0.5, 1, 1.5, 2, 2.5, 3, 3.5, 4 M of glycine betaine, respectively. Lane 1 is the relaxed pAB1. Lane 11 is the DNA sample after the DNA supercoiling assay using 20 nM of *E. coli* DNA gyrase. Gyr_Mtb and Gyr_Ec represent *Mtb* and *E. coli* DNA gyrase, respectively. *Mtb*, *Mycobacterium tuberculosis*.

To study how macromolecular crowding stimulates DNA supercoiling activity of *Mtb* DNA gyrase, we used a buffer solution containing only minimal required components, including 40 mM Tris–HCl pH 7.5, 10 mM MgCl₂, 100 mM KAc, 4 mM DTT, and 2 mM ATP. We also used 5 nM of *Mtb* DNA gyrase, a concentration that did not significantly supercoil Rx pAB1 in the absence of a crowding agent or a stimulating factor (lane 1 of Fig. 1*A*). Since previous studies showed that potassium glutamate greatly stimulates the DNA supercoiling activity of *various* DNA gyrase including *Mtb* DNA gyrase (9, 24, 30), we examined whether potassium glutamate can also stimulate the DNA supercoiling activity of *Mtb* DNA gyrase under our experimental conditions. As expected, 50 to 70 mM of potassium glutamate stimulated the DNA supercoiling activity of *Mtb* DNA gyrase (Fig. 1*A*). Similar results were obtained for *E. coli* DNA gyrases (Fig. 1*B*). These results are consistent with previously published results (9, 24, 30).

We next examine whether glycine betaine (Fig. S1), a small molecule compound found in many species in all domains of life including bacteria (43, 44), can stimulate the DNA supercoiling activity of *Mtb* DNA gyrase. Glycine betaine is often used and accumulated inside bacterial cytosol at high concentrations to protect cells from osmotic stress (45, 46, 47). In this way, the water activity in cell cytosol is greatly reduced and osmotic pressure inside cells is increased correspondingly to balance the osmotic pressure across the two sides of cell membrane. We expect that high concentrations of glycine betaine should greatly stimulate the DNA supercoiling activity. Indeed, results in Figure 1*C* clearly demonstrate that 2 M of glycine betaine significantly stimulated the DNA supercoiling activity of *Mtb* DNA gyrase although the stimulation is diminished at higher concentrations of glycine betaine. These results suggest that the stimulation of *Mtb* DNA gyrase’s DNA supercoiling activity by potassium glutamate and glycine betaine is through reducing the water activity in the aqueous buffer solutions. Similar results were obtained for *E. coli* DNA gyrase (Fig. 1*D*), suggesting that this is a general phenomenon for bacterial DNA gyrase.

**Figure 2:**
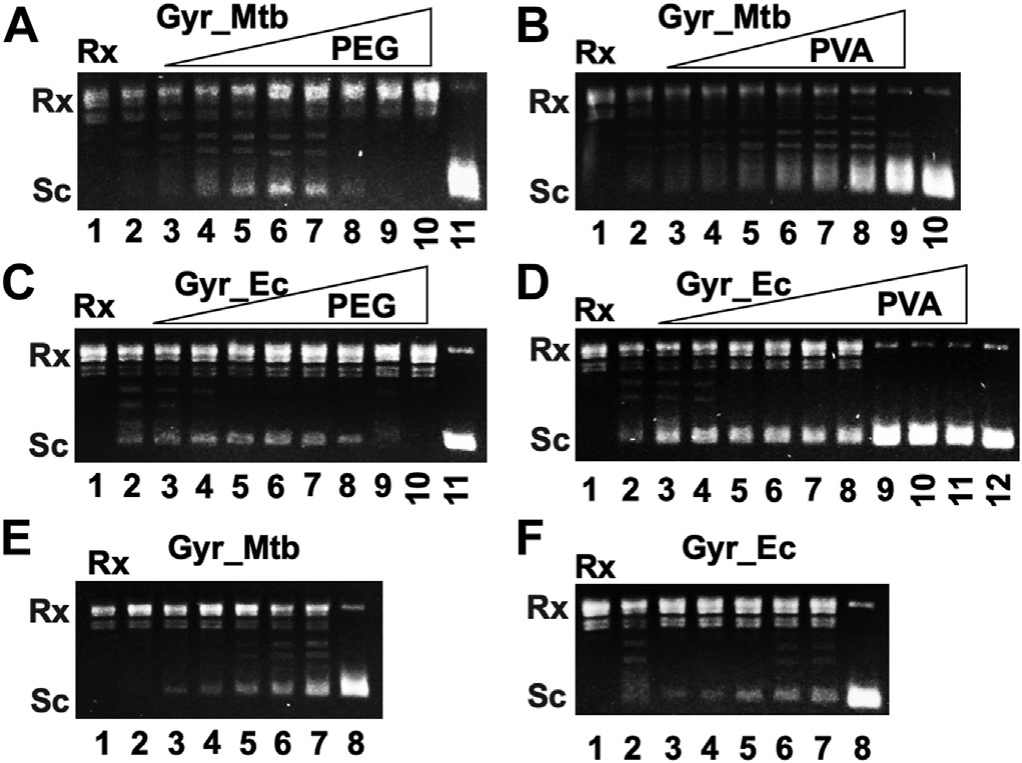
**Stimulation of DNA supercoiling activity of *Mtb* and *Escherichia coli* DNA gyrase by PEGs and PVA.** DNA gyrase supercoiling assays were performed as described under Experimental procedures. Five nanomolars of *Mtb* DNA gyrase or 2 nM of *E. coli* DNA gyrase was used in the assays. The 1× gyrase buffer contains 40 mM Tris–HCl pH 7.5, 10 mM MgCl₂, 100 mM KAc, 4 mM DTT, and 2 mM ATP. *A*, stimulation of the *Mtb* DNA gyrase supercoiling activity by PEG20,000. Lanes 2 to 10 contain 0, 1, 2, 3, 4, 5, 7, 10, and 20% of PEG20,000, respectively. Lane 1 is the relaxed pAB1. Lane 11 is the DNA sample after the DNA supercoiling assay using 20 nM of *Mtb* DNA gyrase. *B*, stimulation of the *Mtb* DNA gyrase supercoiling activity by PVA. Lanes 2 to 8 contain 0, 1, 2, 3, 4, 5, 6, and 7% of PVA, respectively. Lane 1 is the relaxed pAB1. Lane 10 is the DNA sample after the DNA supercoiling assay using 20 nM of *Mtb* DNA gyrase. *C*, stimulation of the *E. coli* DNA gyrase supercoiling activity by PEG20,000. Lanes 2 to 10 contain 0, 1, 2, 3, 4, 5, 7, 10, and 20% of PEG20,000, respectively. Lane 1 is the relaxed pAB1. Lane 11 is the DNA sample after the DNA supercoiling assay using 20 nM of *E. coli* DNA gyrase. *D*, stimulation of the *E. coli* DNA gyrase supercoiling activity by PVA. Lanes 2 to 8 contain 0, 1, 2, 3, 4, 5, 6, and 7% of PVA, respectively. Lane 1 is the relaxed pAB1. Lane 12 is the DNA sample after the DNA supercoiling assay using 20 nM of *E. coli* DNA gyrase. *E*, stimulation of *Mtb* DNA gyrase activity depends on the molecular weight of PEGs. Lanes 2 to 7 contain 5% of PEG400, 1,450, 3,350, 8000, and 20,000, respectively. Lane 1 is the Rx pAB1. Lane 8 is the DNA sample after the DNA supercoiling assay using 20 nM of *Mtb* DNA gyrase. *F*, stimulation of *E. coli* DNA gyrase activity depends on the molecular weight of PEGs. Lanes 2 to 7 contain 5% of PEG400, 1450, 3350, 8000, and 20,000, respectively. Lane 1 is the Rx pAB1. Lane 8 is the DNA sample after the DNA supercoiling assay using 20 nM of *E. coli* DNA gyrase. Gyr_Mtb and Gyr_Ec represent *Mtb* and *E. coli* DNA gyrase, respectively. *Mtb*, *Mycobacterium tuberculosis*; PEG, polyethylene glycol; PVA, polyvinyl alcohol; Rx, relaxed.

PEG and PVA are two polymers widely used in the *in vitro* studies of macromolecular crowding effects on different biological reactions/processes since these two polymers can mimic the macromolecular crowing environment inside cells where high concentrations of macromolecules, such as proteins and nucleic acids, are present (31, 33). We decided to test whether and how PEG and PVA affect the DNA supercoiling activity of *Mtb* DNA gyrase. Figure 2, *A* and *B* show our results. Both PEG20,000 (Fig. 2*A*) and PVA30,000 (Fig. 2*B*) greatly stimulated the DNA supercoiling activity of *Mtb* DNA gyrase. There are some differences between these two polymers. The stimulation of DNA supercoiling activity depended on the concentration of PEG20,000 and reached the highest when 5% PEG20,000 was used (lane 6 of Fig. 2*A*). The stimulation decreased and eventually diminished when higher concentrations of PEG20,000 were used (Fig. 2*A*). In contrast, we only observed the stimulation effects on the DNA supercoiling activity of *Mtb* DNA gyrase for PVA30,000 (Fig. 2*B*). The higher the PVA30,000 concentration the more stimulation (Fig. 2*B*). Eventually, all Rx pAB1 became (−) Sc (lane 9 of Fig. 2*B*). It is likely that this difference stems from the hydroxyl groups of PVA30,000 along the polymer backbone since PEG20,000 does not carry these hydroxyl groups (Fig. S1). Similar results were obtained for *E. coli* DNA gyrase (Fig. 2, *C* and *D*), suggesting that the effects of PEGs and PVA on DNA gyrase are not specific to *Mtb* DNA gyrase. We note that PEGs only slightly stimulated the DNA supercoiling activity of *E. coli* DNA gyrase (compare lane 2 to lanes 5 and 6 of Fig. 2*C*). Chloroquine gels showed that the fast-moving bands in these agarose gels are (−) Sc (Fig. S2).

High concentrations of PEGs and PVA reduce water activity and also increase the excluded volume in the reactions where certain volume is inaccessible to other macromolecules due to the presence of PEGs or PVA (31, 33, 48). Our results using potassium glutamate and glycine betaine clearly showed that the decrease of water activity stimulated the DNA supercoiling activity of *Mtb* DNA gyrase (Fig. 1). We wondered whether the excluded volume also contributed to the stimulation of DNA supercoiling activity of *Mtb* DNA gyrase. To test this, we used five PEGs with different molecular weights in our study. If excluded volume contributed to the stimulation, PEGs with higher molecular weights should stimulate more DNA supercoiling than those with lower molecular weights. Our results showed that PEGs with higher molecular weights indeed stimulated more DNA supercoiling for *Mtb* and *E. coli* DNA gyrase (Figs. 2, *E* and *F* and S3). The higher molecular weight of PEGs the more stimulation was observed (compare lanes 3–7 of Fig. 2, *E* and *F*). These results suggest that the excluded volume by PEGs and PVA30,000 also contributed to the stimulation of DNA supercoiling activity of *Mtb* and *E. coli* DNA gyrase.

**Figure 3:**
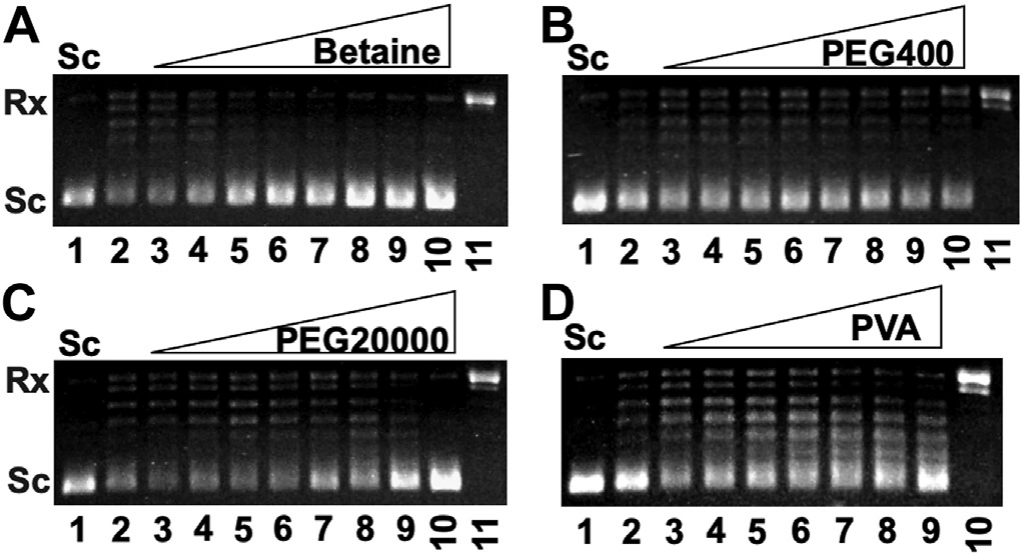
**Effects of glycine betaine, PEG400, PEG20,000, and PVA30,000 to 70,000 on *Mtb* DNA topoisomerase I.** The relaxation assays by *Mtb* DNA topoisomerase I was described under Experimental procedures. Seven nanomolars of *Mtb* DNA topoisomerase I was used in these assays. *A*, effects of glycine betaine on *Mtb* DNA topoisomerase I. Lanes 2 to 10 contain 0, 0.5, 1, 1.5, 2, 2.5, 3, 3.5, 4 M of glycine betaine, respectively. Lane 1 is the relaxed pAB1. Lane 11 is the DNA sample after the DNA relaxation assay using 20 nM of *Mtb* DNA topoisomerase I. *B*–*C*, effects of PEG400 (*B*) and PEG20,000 (*C*) on *Mtb* DNA topoisomerase I. Lanes 2 to 10 contain 0, 1, 2, 3, 4, 5, 7, 10, and 20% of PEG20,000, respectively. Lane 1 is the relaxed pAB1. Lane 11 is the DNA sample after the DNA relaxation assay using 20 nM of *Mtb* DNA topoisomerase I. *D*, PVA’s effects on *Mtb* DNA topoisomerase I. Lanes 2 to 8 contain 0, 1, 2, 3, 4, 5, 6, and 7% of PVA, respectively. Lane 1 is the relaxed pAB1. Lane 10 is the DNA sample after the DNA relaxation assay using 20 nM of *Mtb* DNA topoisomerase I. *Mtb*, *Mycobacterium tuberculosis*; PEG, polyethylene glycol; PVA, polyvinyl alcohol.

We also tested effects of glycine betaine, PEGs, and PVA on *Mtb* DNA topoisomerase I, *E. coli* DNA topoisomerase I, human topoisomerase I, and human DNA topoisomerase IIα. Figure 3 and Fig. S4 show our results. Glycine betaine, PEGs, and PVA did not stimulate the DNA relaxation activities of these DNA topoisomerases. In contrast, they inhibited the DNA relaxation activities of these DNA topoisomerases at high concentrations (Fig. 3 and S4). These results were not unexpected, as assay conditions of low water activities and/or in the presence of macromolecular crowding agents favor more compacted macromolecules (48, 49). (−) Sc DNA is more compacted than Rx DNA, which may partially explain the inhibition of the DNA relaxation activities of these DNA topoisomerases.

### Steady-state kinetics of *Mtb* DNA gyrase in the presence of glycine betaine and PEG400

**Figure 4:**
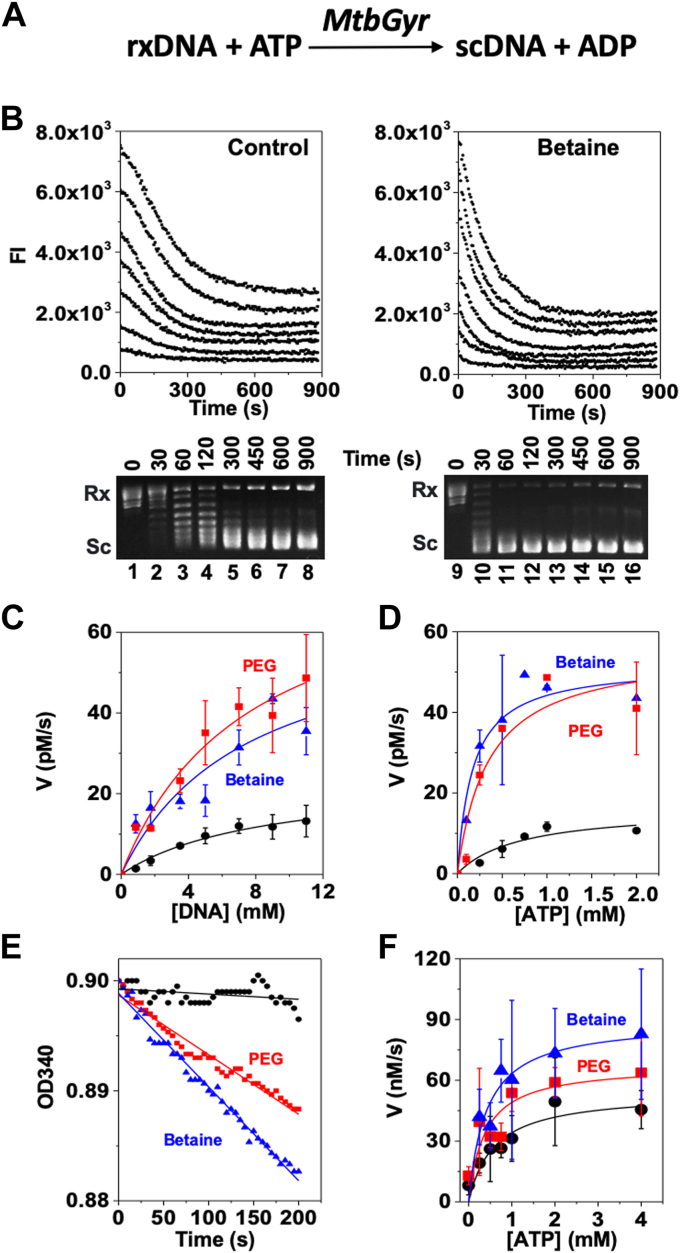
**Steady-state kinetics of *Mtb* DNA gyrase in the absence or presence of glycine betaine or PEG400.***A*, proposed reaction scheme for the supercoiling reaction catalyzed by *Mtb* DNA gyrase. The reaction includes two substrates: ATP and DNA (pAB1_FL924). *B*, time courses of *Mtb* DNA gyrase-catalyzed DNA supercoiling reactions monitored by fluorescence intensity change (*upper*) and agarose gel electrophoresis (*low*). For the supercoiling reaction, 70 μl of 1× DNA gyrase buffer (40 mM Tris–HCl, 10 mM MgCl₂, 100 mM KAc, 4 mM DTT, and 2 mM ATP, pH 7.5) containing different concentrations of rx pAB1_FL924 was prepared and equilibrated to 37 °C, and 50 nM of *Mtb* DNA gyrase was used to supercoil the rx pAB1_FL924. The fluorescence intensity at λem = 582 nm was monitored with λex = 532 nm using a home-made spectrofluorimeter. *C*, initial velocities of supercoiling reaction were calculated from (*B* or Fig. S5), plotted against the substrate (sc pAB1_FL924) concentration, and fitted into the classical Michaelis–Menten equation to determine K_M_, V_max_, and k_cat_ for the DNA substrate (rx pAB1_FL924). *D*, initial velocities of supercoiling reaction were plotted against ATP concentration and fitted into the classical Michaelis–Menten equation to determine K_M_ and V_max_ for ATP. *E*, time courses of *Mtb* DNA gyrase ATPase activities measured by the gyrase ATPase–linked assay as described under Experimental procedures. Glycine betaine and PEG400 greatly stimulated the *Mtb* DNA gyrase ATPase activities. *F*, initial velocities of the gyrase ATPase activities were plotted against ATP concentration and fitted into the classical Michaelis–Menten equation to determine K_M_ and V_max_ for ATP. The standard deviations are calculated according to three independent experiments. *Mtb*, *Mycobacterium tuberculosis*; PEG, polyethylene glycol.

Recently, we developed a supercoiling dependent fluorescence quenching (SDFQ)-based assay to study steady-state kinetics of various DNA topoisomerases including *E. coli* DNA gyrase and successfully determined their steady-state kinetic parameters including K_M_, V_max_, and k_cat_ (50, 51). Here, we carried out similar steady-state kinetic studies for *Mtb* DNA gyrase and determined how glycine betaine and PEG400 affect the K_M_, V_max_, and k_cat_ of this enzyme. Solutions containing other PEGs and PVA are too viscous to prevent them from the SDFQ-based kinetics studies. Figure 4, Fig. S5, and Table 1 summarize our results. Similar to *E. coli* DNA gyrase, addition of *Mtb* DNA gyrase to a reaction containing Rx pAB1_FL924 resulted in a significant decrease of the fluorescence intensity of the solution at 582 nm that reached the plateau around 400 s (Fig. 4). This decrease in fluorescence is indicative of the complete (−) supercoiling of the Rx pAB1_FL924 (Fig. 4*B*). Using these results, we determined the pseudo first-order kinetics for the two substrates of *Mtb* DNA gyrase, Rx pAB1_FL924 (DNA) and ATP, by fitting the initial velocity results to the Michaelis−Menten equation (Fig. 4). In the absence of a stimulating factor, K_M_, V_max_, and k_cat_ of *Mtb* DNA gyrase were determined to be 8.4 ± 3.9 nM, 23.9 ± 6.0 PM/s, and 0.50±0.34 × 10^−3^ s^−1^, respectively for the DNA substrate, Rx pAB1_FL924 (Table 1). K_M_ was determined to be 0.7 ± 0.3 mM for ATP. These results showed that *Mtb* DNA gyrase is slower at supercoiling plasmid DNA and requires higher concentrations of ATP comparing with *E. coli* DNA gyrase under similar experimental conditions (51). These results are also consistent with previously published results (21, 22, 23).

**Table 1:** Steady-state kinetic parameters for *Mtb* DNA gyrase in the absence or presence of PEG400 or glycine betaine

Kinetic parameters	No compound	PEG400 (5%)	Betaine (2 M)
V_max_ (pM/s, DNA)^a^	23.9 ± 6.0	80.5 ± 17.5	64.8 ± 18
k_cat_ (s^−1^ × 10^−3^)^a^	0.5 ± 0.12	1.6 ± 0.35	1.3 ± 0.36
K_M_ (nM, DNA)^a^	8.4 ± 3.9	7.6 ± 3.2	7.5 ± 4
K_M_ (mM, ATP)^a^	0.7 ± 0.3	0.35 ± 0.14	0.17 ± 0.07
Km (mM, ATP)^b^	0.58 ± 0.26	0.38 ± 0.19	0.40 ± 0.22
V_max_ (nM/s, ATP)^b^	53.9 ± 8.5	67.5 ± 9.9	89.2 ± 14.1
k_cat_ (s^−1^ × 10^−3^)^b^	1.1 ± 0.17	1.4 ± 0.2	1.8 ± 0.28
K_d_ (nM)^c^	94 ± 4	60 ± 24	85 ± 6

Abbreviations: GMSA, gel mobility shift assays; *Mtb*, *Mycobacterium tuberculosis*; PEG, polyethylene glycol; SDFQ, supercoiling-dependent fluorescence quenching.

a These kinetic parameters were determined by using SDFQ-based kinetic assays shown in Figure 4, *C*–*E* and Fig. S5.

b These kinetic parameters were determined by using the *Mtb* DNA gyrase ATPase assays shown in Figure 4, *F* and *G*.

c The DNA dissociation constants of *Mtb* DNA gyrase (K_d_) were determined using GMSA assays shown in Fig. S6.

Consistent with agarose gel–based results described above, glycine betaine and PEG400 dramatically changed the kinetics of *Mtb* DNA gyrase, sharply increasing the DNA supercoiling rate of *Mtb* DNA gyrase (Figs. 4 and S5). For example, V_max_ and k_cat_ of *Mtb* DNA gyrase in the presence of PEG400 were determined to be 80.5 ± 17.5 PM/s and 1.6 ± 0.3 S^−1^, respectively, which are approximately three times the values in its absence (Table 1). Similar results were obtained for glycine betaine (Table 1). Interestingly, glycine betaine and PEG400 also significantly decreased the K_M_ values for both substrates: ATP and DNA. For instance, K_M_ of *Mtb* DNA gyrase for ATP was decreased to 0.35 ± 0.14 and 0.17 ± 0.07 mM in the presence of PEG400 and glycine betaine, respectively, which are much lower than the K_M_ value in their absence (Table 1). These K_M_ values are almost the same with those obtained from another assay, the gyrase ATPase linked assay, in which the hydrolysis of ATP by gyrase is linked to the conversion of NADH to NAD^+^ (52) (Table 1). K_M_ for the DNA substrate, pAB1_FL924 was also decreased in the presence of glycine betaine and PEG400 (Table 1), indicating that the gyrase DNA dissociate constant K_d_ should also be decreased. Indeed, our gel mobility shift assays showed that the K_d_ values of *Mtb*–DNA complexes were decreased in the presence of glycine betaine and PEG400 (Fig. S6 and Table 1).

### Molecular dynamics of *Mtb* DNA gyrase in the presence of PEG400

*Mtb* DNA gyrase is a large heterotetramer with a molecular weight of 332,730 Da. The complete tetrameric structure of *Mtb* gyrase-DNA complex is not available. To investigate and compare the role of PEG on the dynamics of GyrB structures, we performed molecular dynamics (MD) simulations for the GyrB–ATP complex as well as the GyrBA–DNA complex in the presence and absence of PEG400.

**Figure 5:**
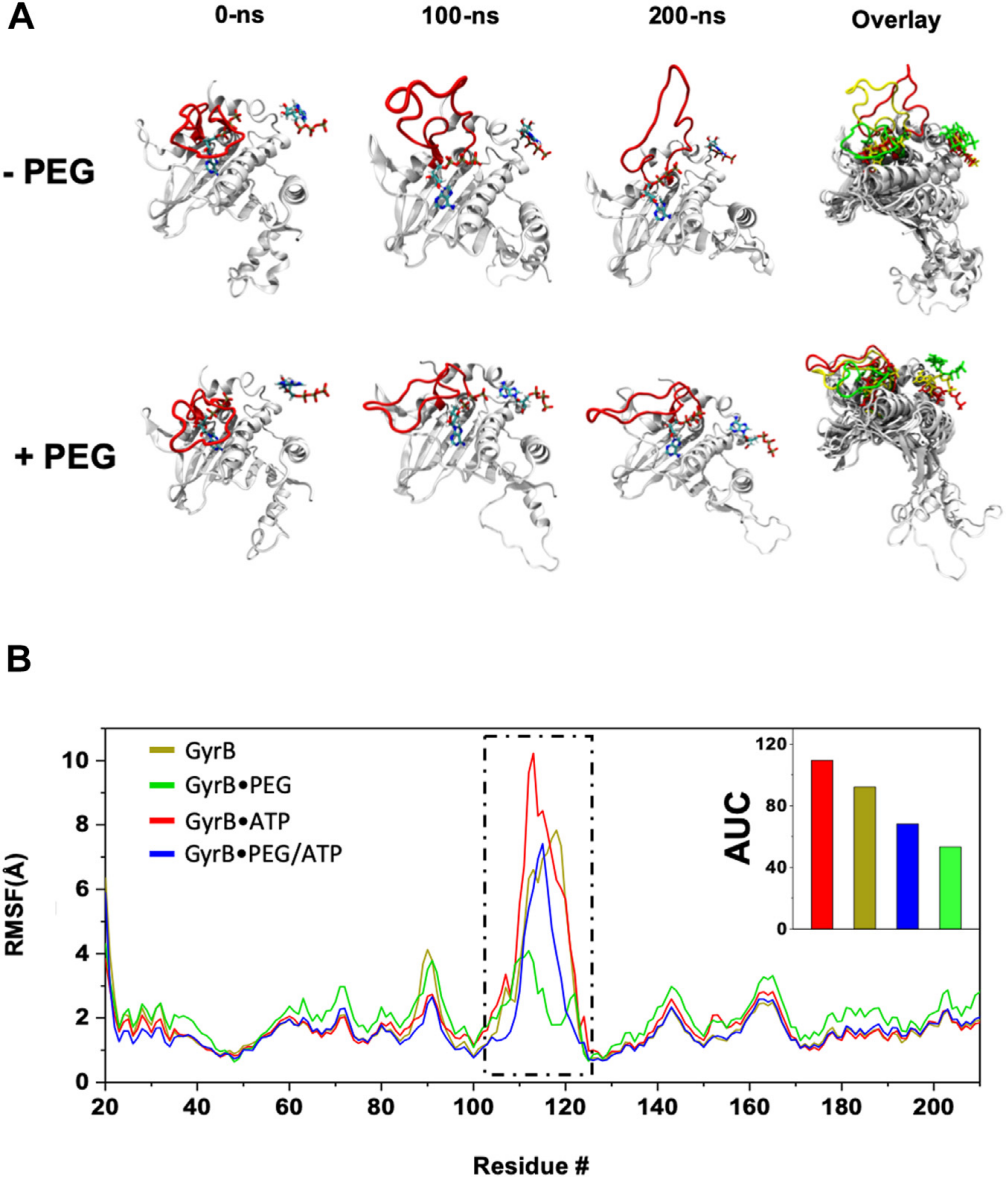
**Molecular dynamic simulation of the *Mtb* GyrB-ATP complex.***A*, the MDS structures of the ATP-binding domain of *Mtb* GyrB at 0, 100, and 200 ns in the absence or presence of PEG400. The structure on the *right side* is the superimposed ATP-binding domains of these three snapshots. The ATP lids (residues 104–125) are highlighted in *red* in the snapshots. For the superimposed structure, the ATP lids are labeled in *green* (0-ns structure), *yellow* (100-ns structure), and *red* (200-ns structure) for comparison. In the absence of PEG, the ATP lid was fully open after 200-ns simulation. *B*, Root-mean-square fluctuations (RMSF) of each amino acid reside in the ATP-binding domain of *Mtb* GyrB. Inset is the histograms for areas under the curve (AUC) measured for the ATP lid (residues 104–125). GyrB, DNA gyrase subunit B; MDS, molecular dynamic simulation; *Mtb*, *Mycobacterium tuberculosis*; PEG, polyethylene glycol.

The dynamics of the GyrB–ATP complex during the 200-ns simulation is shown in Video S1. As expected, no specific interactions between PEG400 and GyrB was found (Fig. S7, *A* and *B*). Interestingly, in the absence of PEG400, we observed a large movement of the ATP lid region, a loop containing amino acid residues 104 to 124 (Fig. 5*A*). The simulation started with the ATP lid in a closed conformation, which likely prevents the ATP from exiting the binding pocket. The loop becomes unstable by 100 ns and fully open by the end of the 200-ns simulation (Fig. 5*A*). The ATP molecule was exposed to the solvent and could depart the binding pocket without a significant energetic barrier. In contrast, in the presence of PEG400, the ATP lid of the GyrB–ATP complex remains in closed or semiclosed conformation (Fig. 5*B*) throughout the simulation. Hydrogen bond analysis (Fig. S8*A*) shows that a salt-bridge between Lys108 in the loop segment and Asp55 contributes the most to the stabilization of the ATP lid in its closed conformation. Other major hydrogen bond pairs include Tyr114–Glu56, Ser117–Asp55, Gly118–Asp55, and Val128–Gly124. The steric repulsions between the ATP lid and PEG400 molecules appear to stabilize the loop in the closed conformation (Fig. 5*B*). This allows the ATP molecule to remain intact in the ATP-binding pocket, increasing the ATP-binding affinity to GyrB. Indeed, the K_M_ value decreased and the ATPase activities of GyrB increased in the presence of PEG400. In the absence of an ATP molecule in the ATP-binding pocket, the ATP lid was observed to be stable in a closed or semiclosed conformation throughout the simulation regardless of the absence or presence of PEG molecules (Fig. S8*B*).

**Figure 6:**
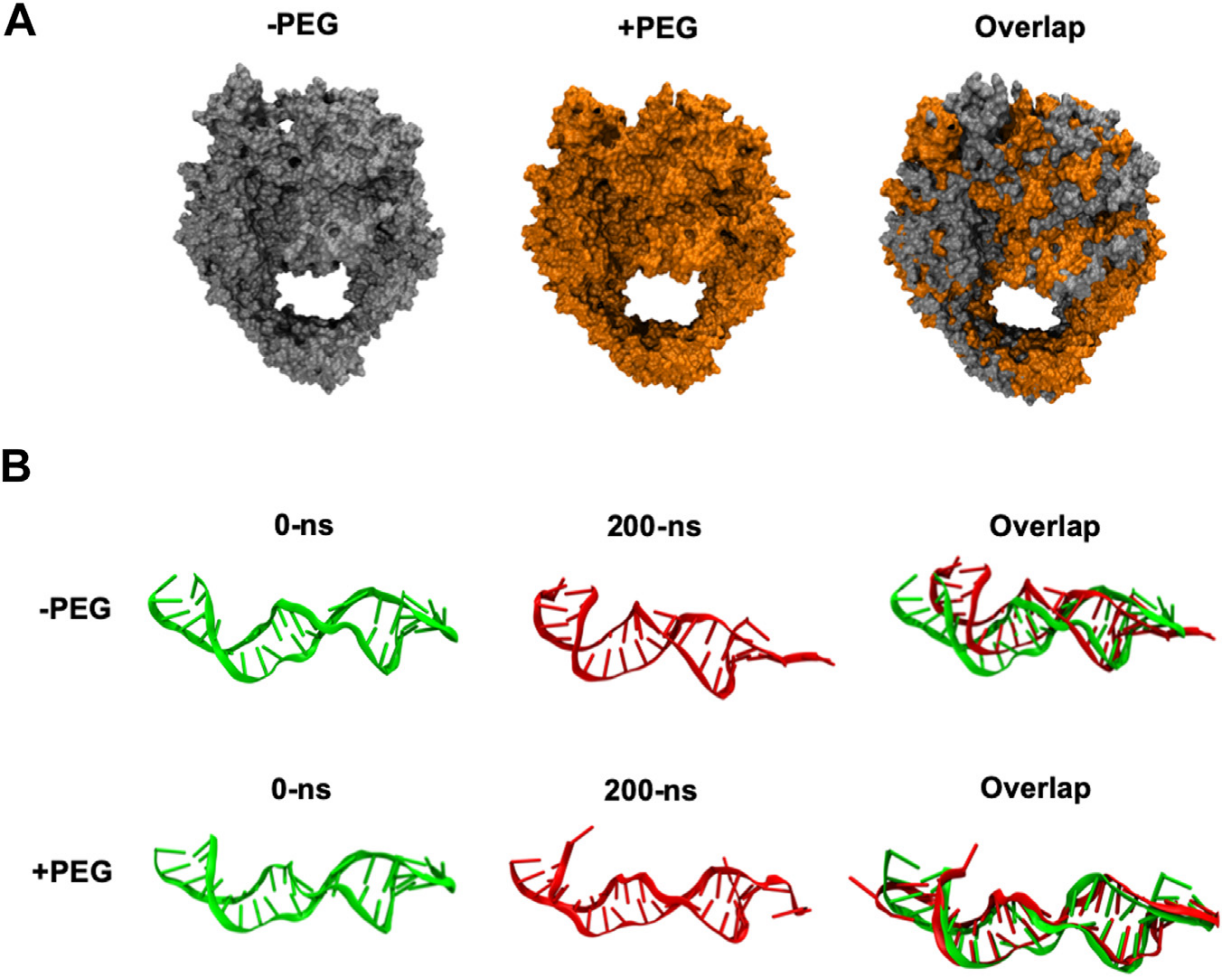
**Molecular dynamic simulation of the GyrBA-DNA complex.***A*, structures of the *Mtb* GyrBA–DNA complex in the absence or presence of PEG400 at the end of 200-ns simulation. The structure on the *right side* is the superimposed structures for comparison. *B*, the DNA structures of the *Mtb* GyrBA–DNA complex in the absence or presence of PEG400 at 0 and 200-ns simulations, respectively. The structures on the *right side* are the superimposed structures for comparison. GyrBA, DNA gyrase subunit BA; *Mtb*, *Mycobacterium tuberculosis*; PEG, polyethylene glycol.

For the GyrBA–DNA complex simulation studies, we used the crystal structure of a *Mtb* GyrBA fusion protein complexed with a 24 bp DNA fragment (PDB ID: 5BS8) (10). We joined the broken DNA strands with covalent bonds so that a full length of the 24 bp DNA fragment was simulated. We placed this modified GyrBA–DNA complex in a cubic water box with TIP3 water and 0.15 M of MgCl_2_ and carried out 200 ns MD simulations in the absence or presence of 180 PEG400 molecules (∼5%). Our results are shown in Figure 6 and Video S2. Again, we did not find direct and specific interactions between the GyrBA–DNA complex and PEG400 molecules (Fig. S7*C*). We also did not observe large-scale conformational changes for the GyrBA–DNA complex during the simulations (Fig. 6). These results are consistent with our CD results (Fig. S9 and Table S1). Some structural changes were observed for the DNA molecule. In the GyrBA–DNA crystal structure complex, the DNA has two upward kinks or bends at both ends due to the intercalation by I181 and I′181 of GyrA (10). After 200-ns simulation in the absence of PEG, one end of the DNA molecule was bent even more due to the fact that I181, but not I′181, moved upward and pushed the DNA end further up (Fig. 6). In contrast, in the presence of PEG, this end of the DNA molecule was straightened due to I181 moving downward. Consequently, the DNA molecule made more contacts with GyrBA (Fig. 6). In summary, in the presence of PEG, the GyrBA–DNA complex was more compact and buried more solvent-accessible surface in the GyrBA-DNA interface (Table S2) compared to the same system in the absence of PEG. This may explain why the gyrase-DNA binding affinity increased in the presence of PEGs or other crowding agents (Fig. S6).

## Discussion

In this article, we conducted a systematic and quantitative study of *Mtb* DNA gyrase under macrocrowding conditions using several molecular crowding reagents, including potassium glutamate, glycine betaine, PEGs, and PVA. These reagents are commonly used in biochemical and biophysical studies to mimic the cellular crowding environment (35, 38, 53). This mimicry is crucial because the cellular interior is densely packed with various macromolecules, such as proteins and nucleic acids (31, 32, 33), significantly influencing biomolecule behavior, including DNA topoisomerases. Our results indeed demonstrated that high concentrations of these molecular crowding reagents significantly stimulated the DNA supercoiling activity of *Mtb* DNA gyrase (Figs. 1 and 2). In contrast, these reagents did not enhance the relaxation activities of other DNA topoisomerases, including *Mtb* DNA topoisomerase I, *E. coli* DNA topoisomerase I, human topoisomerase I, and human DNA topoisomerase IIα (Figs. 3 and S4). At high concentrations, these molecular crowding reagents inhibited the relaxation activities of these DNA topoisomerases (Figs. 3 and S4). Our kinetic results revealed that glycine betaine and PEG400 substantially reduced the K_M_ of *Mtb* DNA gyrase while simultaneously increasing the *k*_*cat*_ of *Mtb* DNA gyrase (Fig. 4 and Table 1). MD simulation studies showed that PEG molecules maintained the ATP lid of GyrB in a closed or semiclosed conformation, preventing ATP molecules from departing the ATP-binding pocket of GyrB (Fig. 5).

We believe that two factors contributed to the stimulation of *Mtb* DNA gyrase under macromolecular crowding conditions: decrease of water activity and increase of excluded volume. The decrease of water activity can be achieved by adding high concentrations of a small molecule osmolyte, such as glycine betaine, and/or a synthetic polymer, such as PEGs or PVA, into an aqueous buffer solution (54, 55), while the increase of excluded volume can only be achieved by adding a high-molecular weight polymer (31, 33). The decrease of water activity is expected to increase the effective concentration of *Mtb* DNA gyrase. The apparent DNA supercoiling activity of *Mtb* DNA gyrase should increase correspondingly. The decrease of water activity also has significant effects on protein–protein, protein–ligand, and protein–DNA interactions (39, 40, 56, 57, 58, 59, 60). Since protein–protein, protein–ligand, and protein–DNA interactions usually result in the burial of hydrophobic, solvent-accessible surfaces at the interaction interfaces (39), the decreased water activity always yields tight bindings for these interactions (39, 40, 56, 57, 58, 59, 60). Indeed, our results showed that the DNA-binding affinity of *Mtb* DNA gyrase was increased in the presence of glycine betaine and PEG400. The K_M_ values of *Mtb* DNA gyrase for both ATP and plasmid DNA pAB1 were also decreased likely due to the increase of binding affinities. These results suggest that the decrease of water activity has a fundamental impact on the *Mtb* DNA gyrase’s DNA supercoiling activity.

Our results clearly demonstrated that the increase of excluded volume greatly stimulated the DNA supercoiling activity of *Mtb* DNA gyrase (Fig. 2). Increasing excluded volume by adding PEGs and PVA to the reaction mixtures is expected to result in steric repulsion between *Mtb* DNA gyrase and these polymers, and form a sterically inaccessible area around the protein, forcing *Mtb* DNA gyrase to occupy a confined volume and limiting its structural freedom (31, 33, 48, 49). As *Mtb* DNA gyrase cannot penetrate the space occupied by PEGs or PVA, this essentially “crowds” the protein, restricting its adopting certain conformations. Our MD simulation studies showed that steric repulsion between *Mtb* GyrB and PEG molecules kept the ATP lid of GyrB in the closed or semiclosed conformation, which prevented ATP molecules departing the ATP-binding pocket (Fig. 5 and Video S1). In this way, the ATP-binding affinity is increased. This can explain our observation by which the K_M_ value of the *Mtb* DNA gyrase ATPase was significantly decreased (Table 1 and Fig. 4), and the V_max_ of the *Mtb* DNA gyrase ATPase was greatly enhanced as well (Table 1 and Fig. 4). The stimulation of *Mtb* DNA gyrase by PEGs and PVA also stems from the fact that (−) Sc plasmid DNA molecules occupy less volume than Rx DNA molecules. According to Le Chatelier’s principle (48, 49), the DNA supercoiling reaction by DNA gyrase should be in favor and stimulated under macromolecular crowding conditions. Furthermore, soft interactions, such as weak hydrogen bonding, between PEGs/PVA and *Mtb* DNA gyrase may also contribute to the stimulation under the crowding conditions (33, 49, 61). The hydroxyl groups of PVA30,000 along the polymer backbone may form many weak hydrogen bonds with *Mtb* DNA gyrase and stabilize its structures/conformations in favor of DNA supercoiling reactions. This may be the reason why we only observed stimulation effects of *Mtb* DNA gyrase by PVA30,000: the higher the PVA concentration the more stimulation (Fig. 2, *B* and *D*). Further studies are needed to fully understand this effect.

## Conclusion

In this paper, we demonstrate that high concentrations of potassium glutamate, glycine betaine, PEGs, and PVA substantially stimulate the DNA supercoiling activity of *Mtb* and *E. coli* DNA gyrase. Steady-state kinetic studies show that glycine betaine and PEG400 greatly reduce K_M_ of *Mtb* DNA gyrase and simultaneously increase V_max_ or k_cat_ of *Mtb* DNA gyrase for ATP and the plasmid DNA molecule. MD simulation studies demonstrate that PEG molecules keep the ATP lid of GyrB in a closed or semiclosed conformation, which prevent ATP molecules from departing the ATP-binding pocket of GyrB. The stimulation of the DNA supercoiling activity of *Mtb* DNA gyrase by these molecular crowding agents is likely coming from the decrease of water activity and the increase of excluded volume.

## Experimental procedures

### Proteins, plasmids, DNA oligomers, and other reagents

Rx plasmids pAB1 (27,357 bp) and pAB1_FL905 or pAB1_FL924 were prepared as described previously (50). Oligonucleotides FL1013 5′-TCAGTCCGACATCGGTCATGAATGACTATGCACGTAAACGAGATGCCAAC-[Tamra∼Q]-3′ and FL1014 5′-GTTGGCATCTCGTTTACGTGCATAGTCATTCATGACCGATGTCGGACTGA-3′ were purchased from Eurofins Genomics, Inc. Plasmids pET28α(+)-His-TEV-*Mtb*-gyrA, pET28α(+)-His-TEV-*Mtb*-gyrB, and pET28α(+)-His-TEV-huTopI were purchased from Gene Universal, Inc (https://www.geneuniversal.com/). PEG 400, PEG 1450, PEG 3350, PEG 8000, PEG 20000, PVA 30,000 to 70,000, and glycine betaine were purchased from Sigma-Aldrich, Inc.

*E. coli* DNA gyrase, *E. coli* DNA topoisomerase I, and His-tagged human DNA topoisomerase IIα C-terminal deletion mutant (hTopo2α-ΔCTD) were purified as described previously (51, 62). *Mtb* topoisomerase I was kindly provided by Prof. Yuk-Ching Tse-Dinh at Florida International University. Pyruvate kinase/lactic dehydrogenase enzymes from rabbit muscle were purchased from Sigma-Aldrich, Inc.

His-tagged *Mtb* gyrase subunit B was expressed and purified from *E. coli* strain BLR(DE3) carrying plasmid pET28α(+)-His-TEV-*Mtb*-gyrB by Ni-NTA column followed by Q Sepharose column. His-tag was removed by tobacco etch virus (TEV) protease digestion. His-tagged *Mtb* gyrase subunit A was purified from *E. coli* strain BLR(DE3) carrying plasmid pET28α(+)-His-TEV-*Mtb*-gyrA by Ni-NTA column. His-tag was also removed by TEV protease digestion. *Mtb* gyrase was reconstituted by mixing gyrA and gyrB at a 1:1 M ratio. The reconstituted *Mtb* gyrase was further purified by size-exclusive chromatography on an FPLC instrument. His-tagged human DNA topoisomerase I was purified using a Ni-NTA column, followed by size-exclusive chromatography on an FPLC instrument from *E. coli* strain BLR(DE3) carrying plasmid pET28α(+)-His-TEV-huTopI. His-tag was also removed by TEV protease digestion.

### DNA supercoiling assays by *E. coli* or *Mtb* DNA gyrase

DNA supercoiling assays were carried out in 30 μl of 1× DNA gyrase buffer (40 mM Tris–HCl, 10 mM MgCl₂, 100 mM KAc, 4 mM DTT, and 1 mM ATP, pH 7.5) using *Mtb* DNA gyrase (30 or 5 nM) or *E. coli* DNA gyrase (20 or 2 nM) and 200 ng of the rx pAB1 in the absence or presence of various concentrations of a crowding agent, such as PEGs, PVA, and glycine betaine. The reaction mixtures were incubated at 37 °C for 15 min. After the reactions were stopped by the addition of 1 μl of stop solution (2% SDS and 200 mM EDTA) followed by phenol extraction, DNA samples were analyzed using 1% agarose gels in 1× TBE buffer. Gel was stained by ethidium bromide and photographed under UV light.

### DNA relaxation assays by different DNA topoisomerases

DNA relaxation assays by *E. coli* DNA topoisomerase I, *Mtb* DNA topoisomerase I, human DNA topoisomerase I, and human DNA topoisomerase IIα was described previously (51). After phenol extraction, DNA samples were analyzed by using 1% agarose gels in 1× TBE buffer, followed by ethidium bromide staining and photographed under UV light.

### Gel mobility shift assay

A 50 bp DNA oligomer, the annealing product of oligonucleotides FL1013 and FL1014 which contains a *Mtb* gyrase preferred binding site, was used in gel mobility shift assays. The gyrase–DNA complexes were formed using 60 nM of the 50 bp DNA oligomer and various amounts of *Mtb* DNA gyrase in 1× DNA binding buffer (30 mM Tris–HCl, 2 mM MgCl₂, 1 mM DTT, 3% glycerol, pH 7.5). If needed, 5% of PEG400 or 2 M glycine betaine was added to the binding reactions. The reaction mixtures were incubated at room temperature for 25 min and loaded to a 10% PAGE gel in 1× TBE buffer, followed by ethidium bromide staining and photographed under UV light. The apparent DNA K was obtained by nonlinear-least-squares fitting the following equation using Origin:

(1) $$R=\frac{\left(a+x+K_{d}\right)+\sqrt{{\left(a+x+K_{d}\right)}^{2}-4ax}}{2a}$$

where a and x represent the total DNA and the total protein concentration, respectively. R is the binding ratio, which is equal to the ratio of the bound DNA divided by the sum of the bound and free DNA.

### Steady-state kinetics of *Mtb* DNA gyrase using SDFQ-based DNA gyrase assays

All SDFQ-based steady-state kinetic measurements of *Mtb* DNA gyrase were performed in 70 μl of 1× gyrase buffer (40 mM Tris–HCl, 10 mM MgCl₂, 100 mM KAc, 4 mM DTT, and 2 mM ATP, pH 7.5), containing rx pAB1_FL924 and 50 nM of *Mtb* DNA gyrase as described previously (51). Briefly, kinetic reaction mixtures were assembled on ice (without *Mtb* DNA gyrase) and equilibrated to 37 °C usually for 5 min (in a cuvette inside the spectrofluorimeter). Then, *Mtb* DNA gyrase was added directly to the cuvette. The fluorescence intensity of the reaction mixture at 582 nm were recorded every 5 s. The initial velocity of the reactions was calculated from linear fitting of the first 5 to 10 data points. The steady-state kinetic parameters K_M_, V_max_, and k_cat_ were obtained by fitting the Michaelis–Menten equation:

(2) $$V_{0}=V_{\max}\frac{\left[S\right]}{K_{M}+\left[S\right]}$$

(3) $$k_{cat}=\frac{V_{\max}}{\left[E\right]}$$

where V_0_, [S], K_M_, V_max_, [E], *k*_*cat*_ represent the initial velocity, substrate concentration, maximum velocity, Michaelis constant, enzyme concentration, and turn-over number, respectively.

### *Mtb* DNA gyrase ATPase assays

DNA gyrase ATPase assays were performed as described previously (52). Briefly, 60 μl of reaction mixtures containing 1×gyrase ATPase buffer containing 50 nM of *Mtb* DNA gyrase, 200 ng of Rx pAB1, 0.8 mM of phosphoenol pyruvate, 1.2 U of pyruvate kinase, 1.7 U of lactate dehydrogenase, and 0.4 mM of NADH were assembled on ice. After the reaction mixtures were incubated 37 °C for 5 min, 2 mM of ATP was added to initiate the reaction. Absorbance at 340 nm was used to monitor the ATPase activities at 37 °C in a spectrophotometer.

### CD spectra

Solutions containing *Mtb* GyrA, GyrB, or gyrase holoenzyme in 1× reaction buffer (40 mM Tris–HCl, 10 mM MgCl_2_, 100 mM KAc, 1 mM β-mercaptoethanol, pH 7.5) were used for CD measurements. CD spectra were recorded at 24 °C on a Jasco J-820 CD Spectrophotometer. The molar ellipticity was calculated from the equation:

(4) $$\left[\theta \right]=\frac{100\theta }{cl}$$

where θ, c, and l are the measured ellipticity in degree, the protein concentration, and the path length, respectively. The CD results were analyzed by using a webserver to obtain different secondary structures for *Mtb* GyrA, GyrB, and gyrase holoenzyme (63).

### MD simulation

All-atom MD simulations were performed for two systems: (1) *Mtb* GyrB’s ATPase domain using the crystal structure of an *Mtb* GyrB–ATP complex as the starting structure (PDB ID:3ZKB) and (2) *Mtb* GyrBA–DNA complex using the crystal structure of the *Mtb* GyrBA fusion protein complexed with a 24 bp DNA fragment and two moxifloxacin molecules (PDB ID: 5BS8). For each of these complexes, simulations were performed with and without PEG. For the PEG systems, 54 molecules of PEG400 (∼5%) were randomly placed in the simulation box. Two ATP molecules were also included (one ATP molecule in the ATP-binding pocket and the other ATP molecule in the solution). Altogether, four GyrB structures were built: GyrB alone, the GyrB–ATP complex, GyrB in the presence of PEG400, and the GyrB–ATP complex in the presence of PEG400. The systems for MD simulations were set up using the CHARMM-GUI web interface (64). Each complex was solvated in a cubic water box with TIP3 water and the system was neutralized by adding 0.15 M of MgCl_2_. All-atom MD simulations were performed with NAMD 2.14 (65) using CHARMM36m (66) force field. The particle mesh Ewald method (67) was used for calculating the long-range ionic interactions. The Nose–Hoover Langevin piston method (68) was used for pressure coupling, with a piston period of 50 fs and a decay of 25 fs, and the Langevin temperature coupling with a friction coefficient of 1 ps^−1^ was used for maintaining the temperature. Each system was minimized for 100,000 steps and equilibrated for 250 ps at 310 K with a 2 fs time step, followed by a 200 ns production simulation performed at a constant pressure of 1 atm and T = 310 K with a 2 fs time step. The solvent-accessible surface area (SASA) was calculated by Visual Molecular Dynamic (69). The fluctuations of the ATP loop were also analyzed by Visual Molecular Dynamic. The burial surface area between the DNA molecule and *Mtb* GyrBA was calculated using the following equation:

(5) $$Burial\;surface\;area=\frac{S_{p}+S_{D}-S_{C}}{2}$$

where *S*_*P*_ and *S*_*D*_ represent the SASA of *Mtb* GyrBA and the DNA molecule, respectively. *S*_*C*_ is the SASA of the *Mtb* GyrBA–DNA complex.

### Molecular docking

AutoDock vina 1.1.2 (https://vina.scripps.edu/) (70) was used to dock the ATP molecule to 1000 conformations generated by MD simulation. The protein pdb files and ATP structure were first converted to pdbqt format for docking. The ATP was screened against the protein conformations using custom scripts, and the resulting scores of the complexes were sorted and ranked according to their binding affinities.

## Data availability

The data that support the findings of this article are available from the corresponding author, F. L., upon request.

## Supporting information

This article contains supporting information.

## Conflict of interest

The authors declare that they have no conflicts of interest with the contents of this article.
