# Supplementary material for: Macromolecular crowding potently stimulates DNA supercoiling activity of Mycobacterium tuberculosis DNA gyrase
Source: J Biol Chem. 2023 Nov 7;299(12):105439. doi: 10.1016/j.jbc.2023.105439 (PMC10731242; doi:10.1016/j.jbc.2023.105439)
Supplement: Supporting Figures S1–S9 and Tables S1 and S2 [file mmc1.pdf]

Macromolecular Crowding Potently Stimulates DNA Supercoiling activity of *Mycobacterium tuberculosis* DNA Gyrase

Zifang Deng[1,2], Prem Chapagain[1,3], and Fenfei Leng[1,2]*
[1]Biomolecular Science Institute, [2]Department of Chemistry & Biochemistry,
[3]Department of Physics, Florida International University, Miami, FL 33199

To whom correspondence should be addressed: Department of Chemistry & Biochemistry,
Florida International University, 11200 SW 8th Street, FL 33199. Tel: 305-348-3277; Fax: 305-348-3772; E-mail: lengf@fiu.edu

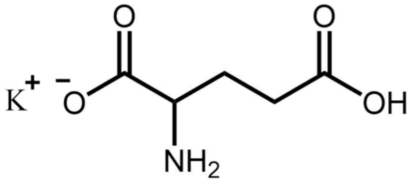

Potassium glutamate

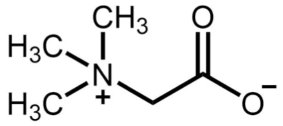

Glycine Betaine

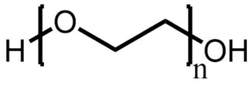

Polyethylene glycol (PEG)

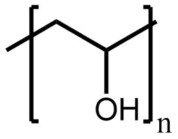

Polyvinyl alcohol (PVA)

Figure S1. Chemical structures of potassium glutamate, glycine betaine, polyethylene glycol (PEG), and polyvinyl alcohol (PVA).

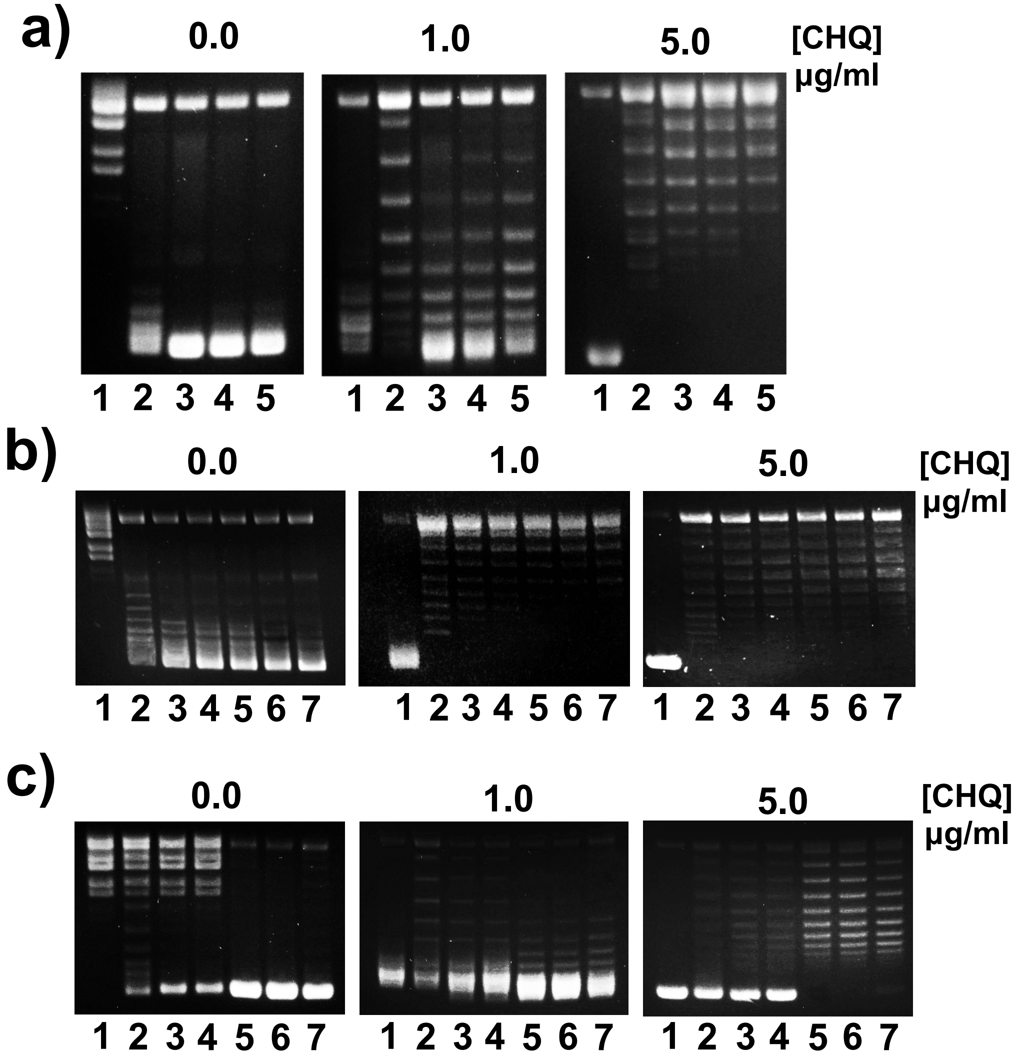

Figure S2. Analysis of DNA topoisomers using 1% agarose gel electrophoreses in the presence of different concentrations of chloroquine to determine the supercoiling density of pAB1 after gyrase supercoiling assays by Mtb or E. coli DNA gyrase in the presence of betaine, PEGs or PVA. a) The supercoiling status of pAB1 after gyrase supercoiling assays by Mtb DNA gyrase in the presence of 2M of glycine betaine (lane 3), 5% of PEG20,000 (lane 4), and 5% PVA (lane 5). Lanes 1 and 2 represent Rx and Sc pAB1, respectively. b and c) The supercoiling status of pAB1 after gyrase supercoiling assays by Mtb (b) and E. coli (c) DNA gyrase in the presence of different concentrations of PVA. Lanes 3-7 contain 5%, 6%, 7%, 8%, and 9% of PVA, respectively. Lanes 1 and 2 represent Rx and Sc pAB1, respectively.

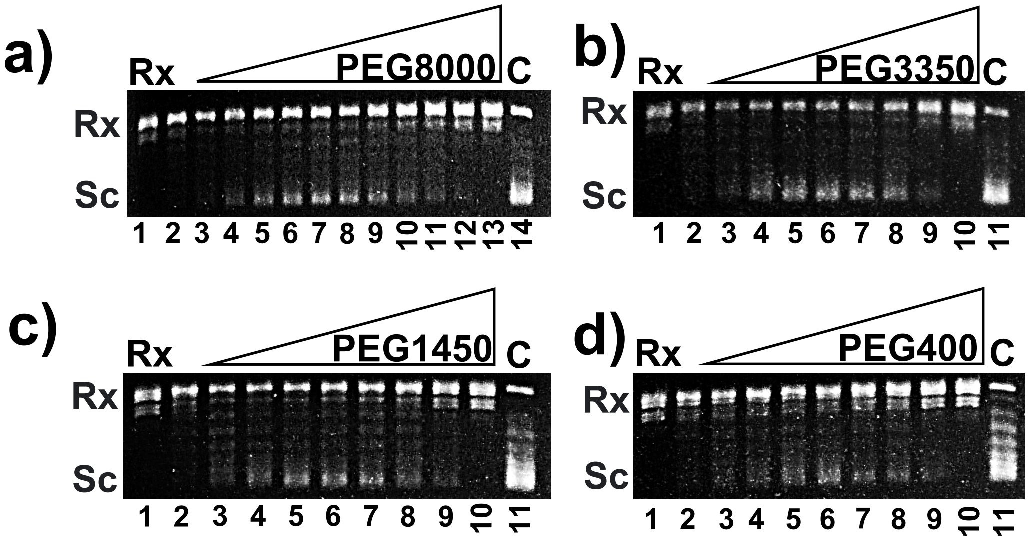

Figure S3. Stimulation of DNA supercoiling activity of Mtb DNA gyrase by PEG400 (a), 1,450 (b), 3,350 (c), and 8000 (d).). DNA gyrase supercoiling assays were performed as described under Materials and Methods. 5 nM of Mtb DNA gyrase was used in the assays. Lanes 2-10 contain 0, 1, 2, 3, 4, 5, 7, 10, and 20% of PEGs, respectively. Lane 1 is the relaxed pAB1. Lane 11?.

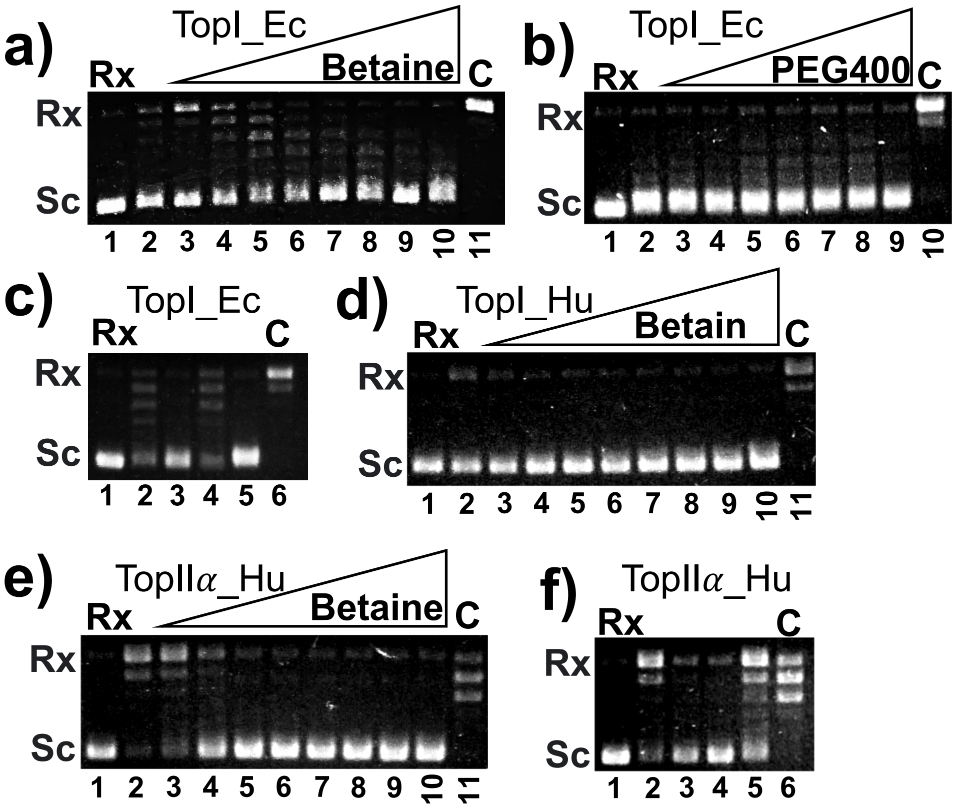

Figure S4. Effects of glycine betaine, PEG400, PEG20,000, and PVA30,000-70,000 on E. coli (a-c) DNA topoisomerase I, human DNA topoisomerase I (d), and human DNA topoisomerase IIα (e and f). The relaxation assays by E. coli DNA topoisomerase I, human DNA topoisomerase I, and human DNA topoisomerase IIa were described under Materials and Methods. a) Effects of glycine betaine on E. coli DNA topoisomerase I. Lanes 2-10 contain 0, 0.5, 1.0, 1.5, 2.0, 2.5, 3.0, 3.5, 4.0 M of glycine betaine, respectively. Lane 1 is the supercoiled pAB1. b) Effects of PEG400 on E. coli DNA topoisomerase I. Lanes 2-10 contain 0, 1, 2, 3, 4, 5, 7, 10, and 20% of PEG400, respectively. Lane 1 is the supercoiled pAB1. c) Effects of 5% of PEG20,000 (lane 3), PEG400 (lane 4), and PVA (lane 5) on E. coli DNA topoisomerase I's relaxation activity. Lane 1 is the supercoiled pAB1. Lane 2 is the relaxation product of pAB1 in the absence of PEGs and PVA. d) Effects of glycine betaine on human DNA topoisomerase I. Lanes 2-10 contain 0, 0.5, 1, 1.5, 2, 2.5, 3, 3.5, 4 M of betaine, respectively. Lane 1 is Sc pAB1. e) Effects of glycine betaine on human DNA topoisomerase IIa. Lanes 2-10 contain 0, 0.5, 1, 1.5, 2, 2.5, 3, 3.5, 4 M of betaine, respectively. Lane 1 is Sc pAB1. f) Effects of 5% of PEG20,000 (lane 3), PEG400 (lane 4), and PVA (lane 5) on human DNA topoisomerase IIa's relaxation activity. Lane 1 is the supercoiled pAB1. Lane 2 is the relaxation product of pAB1 in the absence of PEGs and PVA.

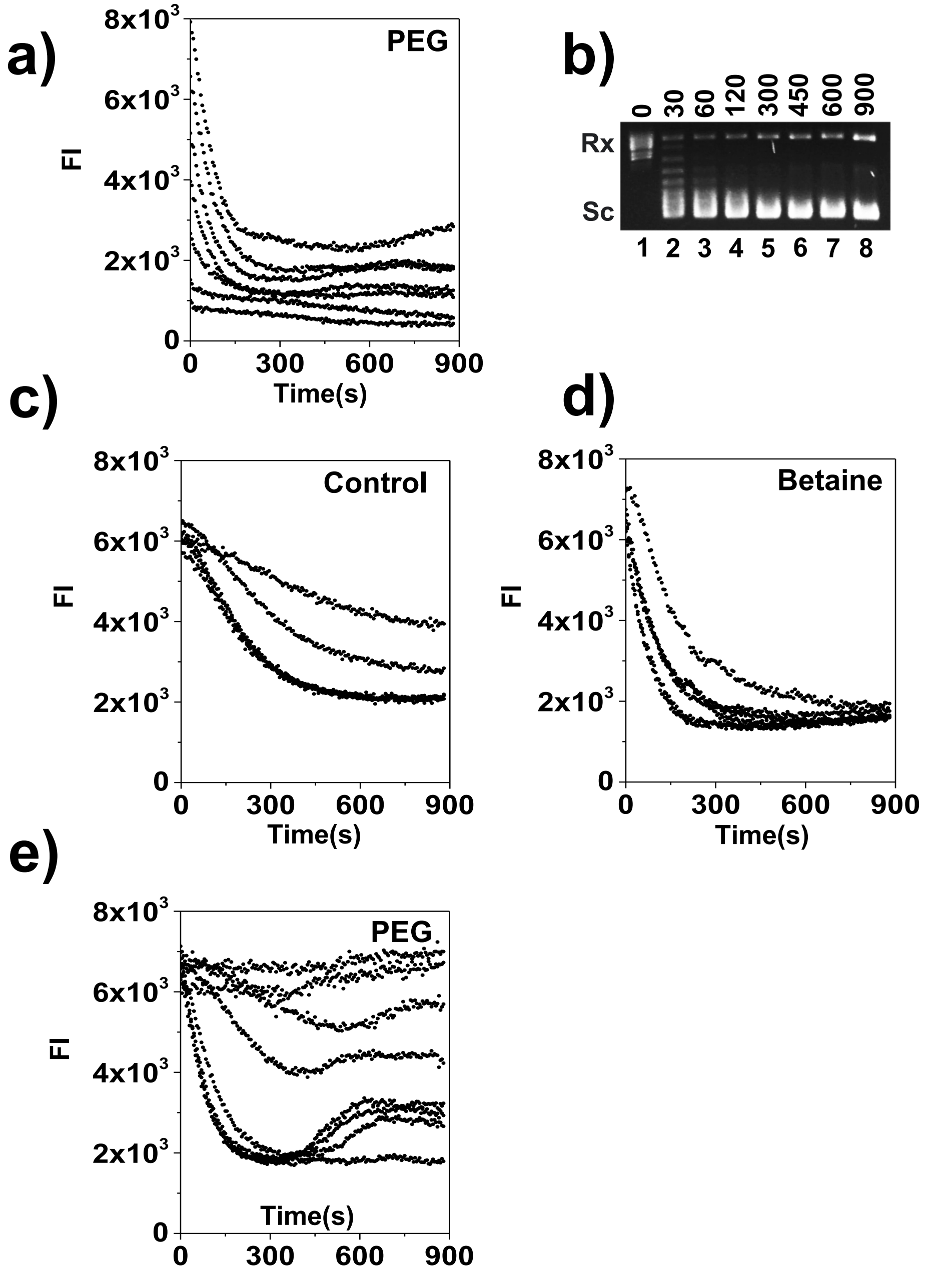

Figure S5. Time courses of DNA supercoiling by Mtb DNA gyrase. a and b) Time course for assays containing different concentrations of rx pAB1_FL924 in the presence of 5% PE. a) SDFQ-based assays. b) Agarose gel-based assays. c-e) Time course for assays containing different concentrations of ATP in the absence © or presence of 2 M glycine betaine (d) or 5% PEG400 (e).

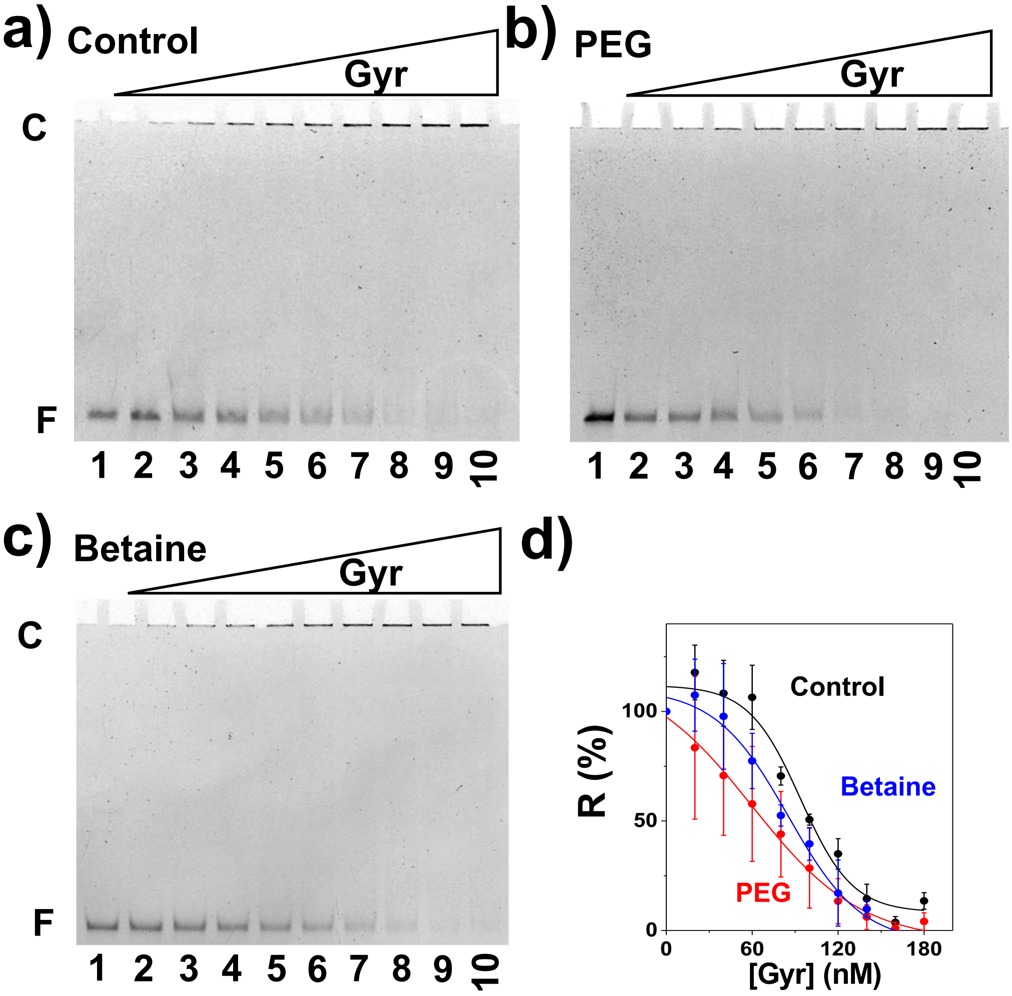

Figure S6. Gel mobility shift assays (GMSAs) to determine the DNA dissociation constants of *Mtb* DNA gyrase binding to a 50-bp DNA oligomer. (**a-c**) GMSAs were performed under Materials and Methods in the absence (**a**) or presence of 5% PEG400 (**b**) or 2 M glycine betaine (**c**). Lanes 1-10 contain 0, 20, 40, 60, 80, 120, 140, 160 and 180 nM of *Mtb* DNA gyrase, respectively. F, free DNA; C, the *Mtb* gyrase-DNA complex. **d**) Quantification analysis of the binding data from the GMSA experiments. The bound ratio of DNA was plotted against the protein concentration. The curves are generated by fitting the data to eq 1 as described under Materials and Methods.

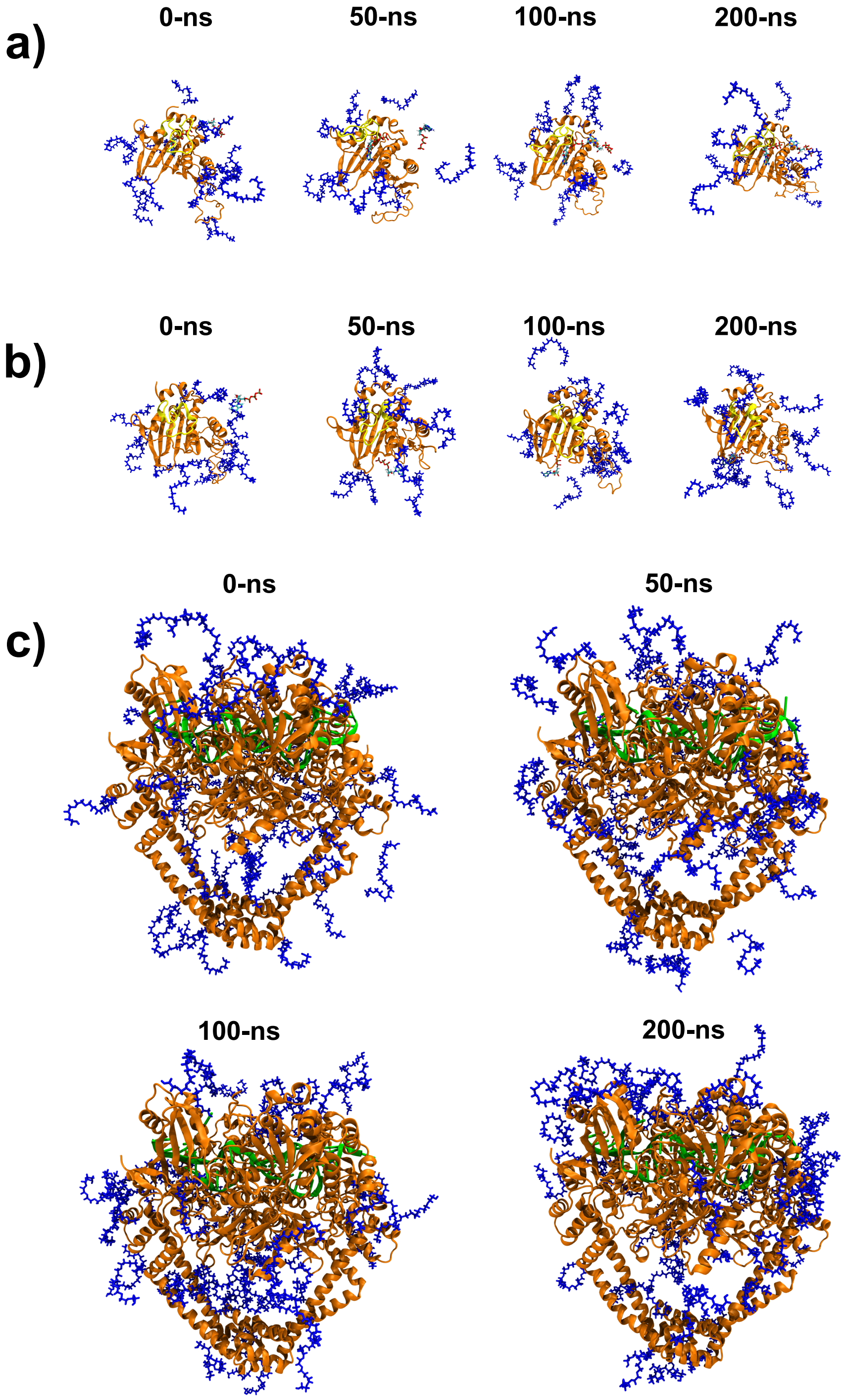

Figure S7. The MDS structures of the ATP binding domain of Mtb GyrB (a (with ATP) and b (no ATP)) and Mtb GyrBA-DNA complex at 0, 50, 100, and 200 ns to demonstrate that PEG400 does not form specific interactions with Mtb DNA gyrase.

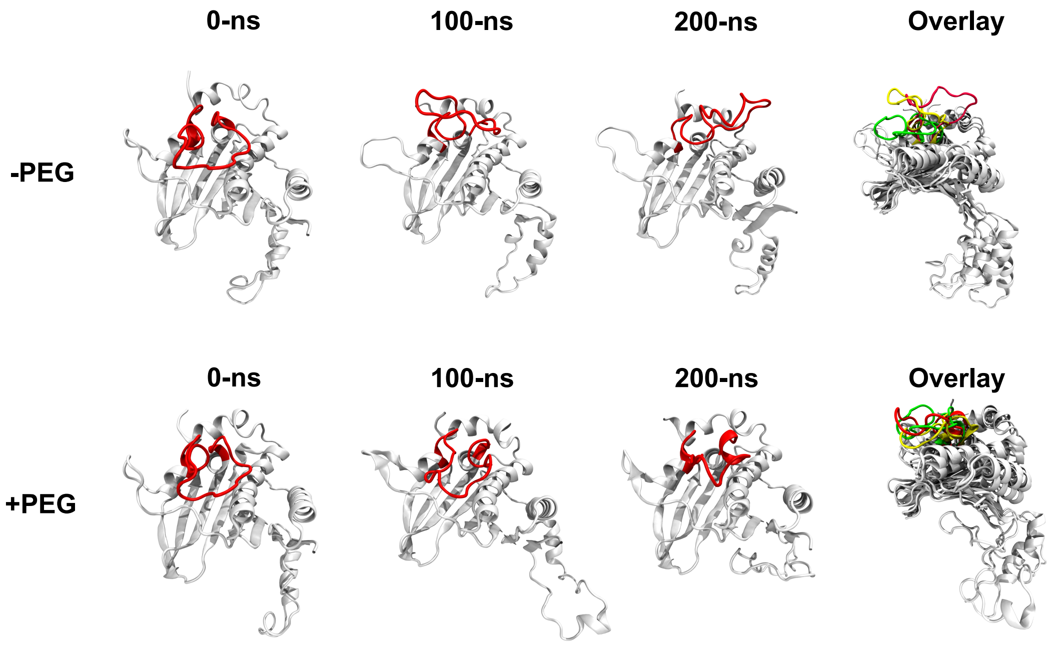

Figure S8. The MDS structures of the ATP binding domain of *Mtb* GyrB at 0, 100, and 200 ns in the absence or presence of PEG400. ATP molecules were not present during the simulations. The structure on the right are the superimposed ATP binding domains of these three snapshots. The ATP lids (residues 104 to 125) are highlighted in red in the snapshots. For the superimposed structure, the ATP lids are labeled in green (0-ns structure), yellow (100-ns structure), and red (200-ns structure) for comparison.

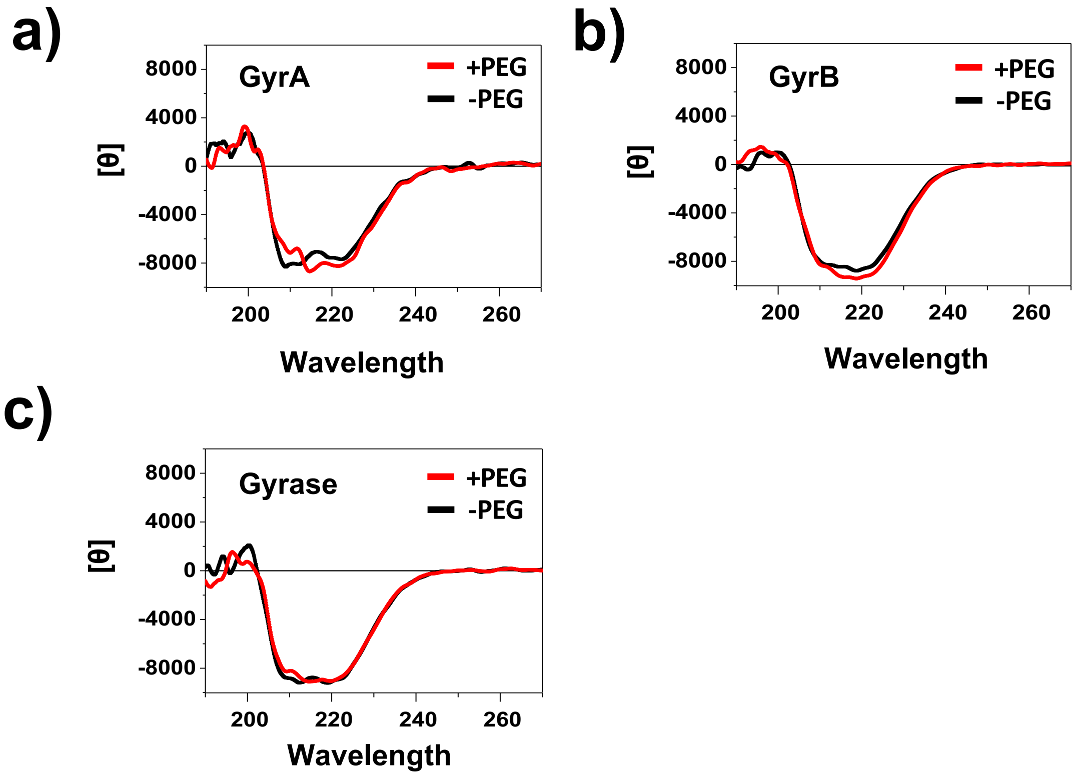

Figure S9. Circular dichroism spectra of Mtb GyrA (a), Mtb GyrB (b), Mtb DNA gyrase holoenzyme (c) in the absence (black) or presence (red) of 5% PEG400.

Table S1. Secondary structure fractions of *Mtb* GyrA, GyrB, and the holoenzyme in the absence or presence of 5% PEG400

		-PEG	+PEG
GyrA	Helix (%)	23.9	9.6
	Antiparallel (%)	18.7	20.5
	Parallel (%)	4.4	12.0
	Turn (%)	16.0	17.6
	Others (%)	37.0	40.3
GyrB	Helix (%)	21.1	18.3
	Antiparallel (%)	17.7	17.7
	Parallel (%)	7.8	10.4
	Turn (%)	13.9	14.0
	Others (%)	39.6	39.6
Gyrase	Helix (%)	21.9	21.4
	Antiparallel (%)	16.4	18.7
	Parallel (%)	5.5	7.9
	Turn (%)	14.3	15.1
	Others (%)	41.9	36.8

The CD results were analyzed at https://bestsel.elte.hu/index.php.

Table S2. The Solvent-Accessible Surface Area (SASA) and burial area of *Mtb* GyrBA-DNA complex after molecular dynamic simulation

	-PEG	+PEG
Mtb GyrBA-DNA complex [Å^2][a]	74875.9	73921.5
Burial area [Å^2][a]	1786.4	2385.3

[a]The SASA and burial areas were calculated as described under Materials and Methods.
